# A Rare Presentation of New-Onset Type 1 Diabetes Mellitus in a Developmentally Delayed Child With an Overlap of Diabetic Ketoacidosis and Hyperglycemic Hyperosmolar State

**DOI:** 10.7759/cureus.28983

**Published:** 2022-09-09

**Authors:** Maria G Parra Villasmil, Shruti Patel, Michael Tansey, Aditya Badheka, Madhuradhar Chegondi

**Affiliations:** 1 Pediatrics, University of Iowa Stead Family Children's Hospital, Iowa City, USA

**Keywords:** diabetes mellitus, adolescent, developmentally delayed children, hyperglycemic hyperosmolar state, diabetic ketoacidosis

## Abstract

Diabetic ketoacidosis (DKA) and hyperglycemic hyperosmolar state (HHS) are serious complications associated with diabetes mellitus (DM). HHS is a common diagnosis in adults but rare in children. DKA is a usual presentation for new-onset type 1 DM, although HHS is rarely a manifestation of new-onset type 1 DM. Diagnosis and management of HHS are challenging in pediatric patients, especially if they present with a mixed picture of HHS and DKA. We report an adolescent female with a new onset of type 1 DM presented as mixed DKA and HHS. Treatment included meticulous management of fluids and continuous insulin drip with the resolution of acidosis within 24 hours and hyperosmolar state at 96 hours of admission. Early differentiation of these two entities and meticulous fluid management improves the outcome and decreases the risk of complications such as cerebral edema, renal failure, and thrombosis, among others.

## Introduction

Diabetes mellitus (DM) in youth presents a substantial clinical and public health burden owing to challenges of disease management and the risk of acute and chronic complications. The incidence of type 1 DM has increased worldwide in the last few decades, with 21.7 cases per 100,000 youths per year in 2011-2012 [1], and 22.3 per 100,000 in 2014-2015 [2]. DM presents as diabetic ketoacidosis (DKA) in 15-70% of newly diagnosed cases in children and adolescents in Europe and North America [3], and 28% in the SEARCH for diabetes in youth study [4]. The incidence of HHS is increasing, with a reported incidence of 2% of youth with type 2 DM at presentation [3].

Hyperglycemic hyperosmolar state (HHS) is a rare complication of DM, characterized by plasma glucose concentration of >600 mg/dL, venous pH >7.25, or serum bicarbonate >15 mmol/L, mild to absent ketonemia, effective serum osmolality >320 mOsm/kg, and altered consciousness (obtundation, combativeness, seizures) [3]. The literature for HHS in children is mainly limited to case series, with a reported mortality of 24% and residual neurological deficits in 35-50% of cases [5]. DKA is a diagnostic triad of hyperglycemia (glucose >200 mg/dL), metabolic acidosis with venous pH <7.3, or serum bicarbonate <15 mmol/L, and ketonemia (ß-hydroxybutyrate ≥3 mmol/L) or moderate to large ketonuria [3]. DKA management requires high doses of insulin with slow fluid replacement to avoid potential cerebral edema, a complication with 0.3-1% incidence and 20-40% mortality [6]. Rapid fluid administration with abrupt changes in serum osmolality is the proposed mechanism for the development of cerebral edema. In contrast, HHS is managed with aggressive fluid resuscitation and electrolyte replacement to replenish fluid losses [3]. A formal distinction between HHS and DKA exists, but diagnosis and management become challenging when the presentation is mixed.

Most case reports in children indicate an association of HHS with type 2 DM with obesity or large consumption of carbohydrate-based drinks, as obesity can mask dehydration and delayed presentation. Rosenbloom AL [7], reported 74% of type 1 DM patients with HHS had a body mass index (BMI) <25 kg/m^2^, and 90% of type 2 DM patients with HHS had BMI >25kg/m^2^. The existing literature on the association of HHS combined with type 1 DM is limited to a few case reports and small case series. However, the literature typically reports HHS without DKA overlap and normal or low sodium levels [8-10]. We report an adolescent female with new-onset type 1 DM presenting with mixed features of DKA and HHS.

This article was previously presented as a poster at the 50th Society of Critical Care Medicine Annual Conference, Anaheim, CA, in February 2021. It was a virtual event given the coronavirus disease 2019 (COVID-19) pandemic.

## Case presentation

A 14-year-old female with significant cognitive and physical disabilities presented with four weeks of worsening irritability, polyuria, and polydipsia, and two days of lethargy and reduced oral intake. The patient had no recent history of fever, rhinorrhea, diarrhea, vomiting, or seizures. Her past medical history was significant for long-standing cognitive delays due to neonatal insults as well as spastic quadriplegia, epilepsy, congenital hydrocephalus with a ventriculoperitoneal (VP) shunt, and failure to gain weight for the last year despite increasing calorie intake. At baseline, the child is non-verbal but interactive and exhibits emotions through laughter and crying. She was taking a soft diet orally with an inability to feed herself independently and no access to free water. She has well-controlled epilepsy with levetiracetam and lamotrigine but does not take any further medications for other co-morbidities. She had a family history of type 2 DM among multiple members and hypothyroidism in her mother.

On admission to the Pediatric Intensive Care Unit, she was afebrile, with respiratory rate 29/min, heart rate 164/min, blood pressure 54/40 mm Hg, and oxygen saturation 91% at room air. She was somnolent but arousable with dry mucous membranes, decreased skin turgor, and capillary refill time >three seconds, suggesting severe dehydration with decompensated shock. Admission weight was 22 kg (BMI for age <1st percentile), decreased from 29.2 kg a month prior. The growth chart revealed a weight plateau in the last year (decreasing Z-score from -3.78 to -5.9).

Laboratory tests at presentation included: serum glucose 1188 mg/dL, serum osmolarity 428 mOsm/kg, venous blood gas pH 7.09, base excess negative 15 mEq/L, anion gap 31 mEq/L, serum bicarbonate 12 mEq/L, serum sodium 160 mEq/L (corrected sodium 177 mEq/L), hemoglobin (Hb)A1c 13.7%, blood urea nitrogen (BUN) 49 mg/dL and serum creatinine 1.7 mg/dL. Urinalysis demonstrated a specific gravity of >1.030, glucose 3+, and ketone 1+. Additional admission and 24-hour laboratory tests are presented in Table 1.

**Table 1:** Laboratory results showing blood gas, electrolytes, BUN, and serum creatinine at the time of hospitalization and 24 hours after hospitalization. BUN: Blood urea nitrogen

Laboratory variable	On admission	24 hours post hospitalization	Reference ranges
pH	7.09	7.41	7.30 - 7.40
PCO2 (mmHg)	51	37	32 - 45
Serum Bicarbonate (mEq/L)	12	24	22 - 29
Base Excess (mEq/L)	-15	-1	-2 -2
Serum Sodium (mEq/L)	160	167	135 - 145
Serum Potassium (mEq/L)	6.1	3.7	3.5 - 5
Serum Magnesium (mg/dL)	1.5	2.0	1.5 – 2.9
Serum BUN (mg/dL)	49	30	10 - 20
Serum Creatinine (mg/dL)	1.7	0.7	0.4 – 0.9
Serum Blood Glucose (mg/dL)	1188	328	65-139
Serum Osmolarity (mOsm/kg)	428	365	275 - 295

The evaluation suggested HHS with severe metabolic acidosis, including a mixed picture of HHS and DKA and acute kidney injury (AKI). Liver function, coagulation profile, cortisol, adrenocorticotropic hormone (ACTH), and thyroid function tests were normal (see values in Table 2). The COVID-19 test was negative. Ventriculoperitoneal (VP) shunt series was normal on admission. 

**Table 2:** Laboratory values including HbA1c, ketonemia, antibodies, thyroid function test, and cortisol axis for the patient and normal reference range HbA1c: Hemoglobin A1C; GAD: Glutamic acid decarboxylase; IA-2: Islet antigen 2; Ig: Immunoglobulin; TSH: Thyroid-stimulating hormone; T4: Thyroxine; ACTH: Adrenocorticotropic hormone

Laboratory variable	Values	Reference range
HbA1c %	13.7	4.8 -6 %
C-Peptide (ng/mL)	0.3	1.1-4.4
Beta-hydroxybutyric acid (mEq/L)	2.7	0.0-0.3
GAD antibodies (IU/mL)	99.3	0- 5
Insulin Antibodies (U/mL)	3.4	0-0.4
IA-2 Antibodies (U/mL)	<5.4	0-7.4
Islet Cell Antibody IgG	< 1: 4	< 1: 4
IgA (mg/dL)	155	47-249
TSH (µIU/mL)	1.22	0.27-4.20
Free T4 (ng/dL)	0.81	0.90-1.70
ACTH (pg/mL)	5	6-55
Serum Cortisol (µg/dL)	27.6	6.0 - 18.4

Upon arrival at the hospital, the patient received a normal saline (NS) bolus at 20 mL/kg, which was repeated in the emergency department. In the first 36 hours, she received fluid resuscitation with NS and lactated Ringer’s for a total of 100 mL/kg and continuous epinephrine infusion for decompensated shock for the first 48 hours of hospitalization. Epinephrine drip was titrated up to 0.06 mcg/kg/min to maintain mean arterial pressure within a goal for age. Due to severe dehydration, aggressive intravenous hydration therapy was initiated with half-normal saline at two times maintenance. Per DKA protocol, two bag system was used with half-normal saline and a mix of half normal saline and 10% dextrose, as well as potassium chloride and potassium phosphate, these two bags were titrated to maintain glucose concentration between 100-200 mg/dl. Continuous intravenous insulin infusion was started at 0.1 units/kg/hour, per DKA protocol, and decreased within six hours of hospitalization due to acidosis correction and decreasing glucose levels. Given the persistently altered sensorium, on day three of admission, magnetic resonance imaging and magnetic resonance venography of the brain ruled out structural changes and venous sinus thrombosis. 

**Figure 1:**
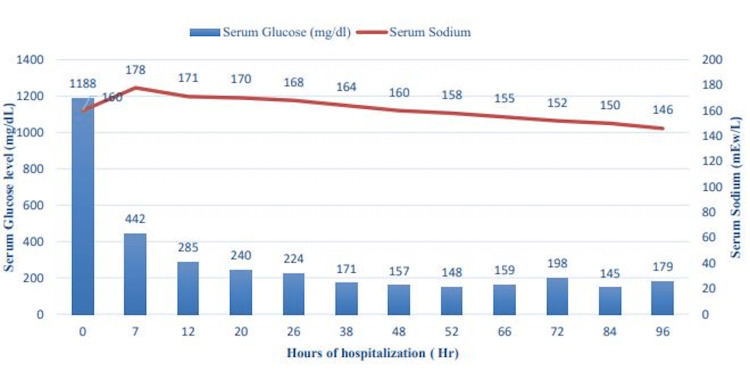
Change of serum glucose level and serum sodium level during first 96 hours of hospital stay.

DKA resolved within 24 hours of hospitalization. HHS correction took over 96 hours, with gradual improvement in sensorium, serum sodium, and glucose (Figure 1). Insulin infusion was transitioned to a subcutaneous insulin regimen on hospital day three after acidosis resolved, and nutrition was initiated via nasogastric tube. Feeds were advanced over the next 48 hours to achieve her caloric goal without evidence of refeeding syndrome. Intravenous (IV) fluids were weaned to maintenance until hyperosmolarity resolved on hospital day four, and the primary team assured that patient was tolerating enteral feeds.

She was diagnosed with type 1 DM with elevated glutamic acid decarboxylase (GAD) and insulin antibodies. She was discharged nine days after admission on multiple daily doses of subcutaneous insulin and a constant carbohydrate liquid diet with nasogastric feedings. Her insulin regimen included glargine insulin 6 units every night, and insulin lispro 6 units four times a day, given with each nasogastric feed, for insulin to carbohydrate ratio per meal of one unit for every 10 grams of carbohydrate (total daily insulin dose was calculated at 1 unit/kg/day).

## Discussion

Our patient presented with an overlap of DKA and HHS in the setting of severe hypernatremic dehydration. This mixed clinical picture made the management challenging. The mortality rate with HHS is as high as 20%, 10 times higher than DKA [11,12], therefore, early detection and intervention are critical.

The classic symptoms of diabetes in children who are non-verbal, for example, younger or developmentally delayed children, can be subtle and hard to recognize. Once a child is verbal and toilet-trained, their frequent requests to get beverages, or use the bathroom may be more likely to be noted by caregivers. Typically, non-verbal children have a delayed diagnosis of diabetes, as they are not able to communicate thirst, and they do not have free access to fluid [13].

DKA is a common presentation in type 1 DM, and HHS is a rare pediatric complication of type 2 DM. Our index case was diagnosed with type 1 DM given low c-peptide and positive glutamic acid decarboxylase and insulin antibodies, despite having a strong family history of type 2 DM. 

DKA and HHS occur due to a critical deficit of insulin action associated with the release of counterregulatory hormones including glucagon, catecholamines, cortisol, and growth hormone resulting in hyperglycemia. HHS facilitates inadequate glucose utilization by insulin-sensitive tissues but is sufficient to prevent lipolysis and ketogenesis. HHS commonly occurs after prolonged and gradually increasing polyuria and polydipsia, resulting in profound dehydration with fluid losses estimated to be twice in patients with HHS compared to DKA. Hypertonicity during the hyperosmolar state preserves intravascular volume, masking the dehydration clinical signs [7]. Higher hepatic and circulating insulin concentration and lower glucagon are present in HHS compared with DKA, which prevents ketogenesis and the development of ketoacidosis in patients with HHS [11].

A more severe degree of electrolyte loss is noticed in HHS in contrast to DKA, given more drastic osmotic diuresis. With massive osmotic diuresis in the early hours of therapy, aggressive fluid is required to reverse shock and avoid complications of vascular collapse (lactic acidosis, rhabdomyolysis, renal failure, thrombosis) [6,14]. On the contrary, DKA is characterized by severe depletion of water and electrolytes from both intra- and extracellular fluid compartments. Despite dehydration, patients generally have normal or even elevated blood pressure [7]. In the current case, the patient presented with decompensated shock, requiring a pressor drip with epinephrine, which resolved within 48 hours. 

Overlap of features between HHS and DKA may occur. Some patients with HHS, especially when severely dehydrated, have mild or moderate acidosis that is mainly due to hypoperfusion and lactic acidosis [3]. As reported by Kershaw et al., 2.5% of 121 children with DM presented with HHS, and only two developed DKA with HHS [15]. Agrawal et al. studied 411 hyperglycemic emergencies in pediatric patients between 2009-2014 and reported 13.8% of patients with a mixed HHS-DKA presentation [16]. McDonell et al. reported five pediatric patients with type 1 DM presenting with hyperosmolar and hypernatremia secondary to excessive consumption of carbonated beverages (5-12 liters) and ‘isotonic’ sports drinks [17], which was not the case in our patient. 

In HHS, ongoing oral fluid intake may delay presentation to medical attention, as it maintains the intravascular volume whereas total body water continues to diminish [3]. Severe hypernatremic dehydration in our index case (estimated fluid deficit of 23%) was likely due to osmotic diuresis and inadequate oral hydration secondary to neurological comorbidity and the inability to express thirst or access to fluid independently. 

In our case, the initial phase of fluid resuscitation was administered using normal saline but was supplemented with lactated Ringer’s to decrease sodium load. In DKA, in addition to a continuous insulin drip, a two-bag system is the standard method of practice including one bag of normal saline and another bag of a mix of NS and 10% dextrose, allowing for titration of glucose infusion as needed. For the treatment of HHS, no specific protocol is available. Often patients need two or three times maintenance fluids based on the level of dehydration and osmolality. Fluid resuscitation with large volumes is not typically seen in patients with DKA, but a hybrid approach was attempted for this patient considering the unusual presentation. For maintenance fluid therapy, we used half-normal saline, at two times maintenance given the degree of free water deficit. However, according to a randomized trial, neither the rate of administration nor the sodium chloride content of intravascular fluids significantly influences the neurological outcomes in children with DKA [18]. Fluid and insulin drip was managed following DKA protocol in our hospital, as well as ISPAD Clinical Consensus Guidelines [3].

Patients with HHS are vulnerable to a number of serious complications, including severe electrolyte imbalances, thrombosis, cerebral edema, malignant hyperthermia, rhabdomyolysis, renal failure, and pancreatitis [7]. Fortunately, for our patient, AKI resolved with fluid resuscitation, and her clinical status improved without any other complications. 

## Conclusions

Children with cognitive impairment can be at risk for severe dehydration due to their inability to access free water and feed independently, especially in the setting of new-onset diabetes. In our index case, severe hyperglycemia, and dehydration in the setting of new-onset type 1 DM resulted in a mixed clinical picture of DKA with HHS. Due to severe dehydration and hypernatremia, the hyperosmolar state was prolonged, although DKA resolved quickly. Prompt recognition of diabetes symptoms in the pediatric population, especially in children with cognitive disabilities, is vital to avoid acute complications including DKA and severe dehydration. Also, meticulous fluid management improves the outcome and decreases the risk of complications.

## References

[1] Mayer-Davis EJ, Lawrence JM, Dabelea D (2017). Incidence trends of type 1 and type 2 diabetes among youths, 2002-2012. N Engl J Med.

[2] Divers J, Mayer-Davis EJ, Lawrence JM (2020). Trends in incidence of type 1 and type 2 diabetes among youths - selected counties and Indian reservations, United States, 2002-2015. MMWR Morb Mortal Wkly Rep.

[3] Wolfsdorf JI, Glaser N, Agus M (2018). ISPAD Clinical Practice Consensus Guidelines 2018: diabetic ketoacidosis and the hyperglycemic hyperosmolar state. Pediatr Diabetes.

[4] Duca LM, Reboussin BA, Pihoker C (2019). Diabetic ketoacidosis at diagnosis of type 1 diabetes and glycemic control over time: the SEARCH for diabetes in youth study. Pediatr Diabetes.

[5] Watanabe S, Kido J, Ogata M, Nakamura K, Mizukami T (2019). Hyperglycemic hyperosmolar state in an adolescent with type 1 diabetes mellitus. Endocrinol Diabetes Metab Case Rep.

[6] Kitabchi AE, Umpierrez GE, Miles JM, Fisher JN (2009). Hyperglycemic crises in adult patients with diabetes. Diabetes Care.

[7] Rosenbloom AL (2010). Hyperglycemic hyperosmolar state: an emerging pediatric problem. J Pediatr.

[8] Kim HJ, Kim DH, Jun YH, Lee JE (2014). A rare diabetes ketoacidosis in combined severe hypernatremic hyperosmolarity in a new-onset Asian adolescent with type I diabetes. BMJ Case Rep.

[9] Basso A, Dalla Paola L, Erle G, Nacamulli D, Armanini D (1997). Hyperosmolar nonketotic coma at the onset of type I diabetes in a child. J Endocrinol Invest.

[10] Murthy S, Sharara-Chami R (2010). Aggressive fluid resuscitation in severe pediatric hyperglycemic hyperosmolar syndrome: a case report. Int J Pediatr Endocrinol.

[11] Pasquel FJ, Umpierrez GE (2014). Hyperosmolar hyperglycemic state: a historic review of the clinical presentation, diagnosis, and treatment. Diabetes Care.

[12] Adeyinka A, Kondamudi NP (2022). Hyperosmolar Hyperglycemic Syndrome. StatPearls [Internet]..

[13] Quinn M, Fleischman A, Rosner B, Nigrin DJ, Wolfsdorf JI (2006). Characteristics at diagnosis of type 1 diabetes in children younger than 6 years. J Pediatr.

[14] Canarie MF, Bogue CW, Banasiak KJ, Weinzimer SA, Tamborlane WV (2007). Decompensated hyperglycemic hyperosmolarity without significant ketoacidosis in the adolescent and young adult population. J Pediatr Endocrinol Metab.

[15] Kershaw MJ, Newton T, Barrett TG, Berry K, Kirk J (2005). Childhood diabetes presenting with hyperosmolar dehydration but without ketoacidosis: a report of three cases. Diabet Med.

[16] Agrawal S, Baird GL, Quintos JB, Reinert SE, Gopalakrishnan G, Boney CM, Topor LS (2018). Pediatric diabetic ketoacidosis with hyperosmolarity: clinical characteristics and outcomes. Endocr Pract.

[17] McDonnell CM, Pedreira CC, Vadamalayan B, Cameron FJ, Werther GA (2005). Diabetic ketoacidosis, hyperosmolarity and hypernatremia: are high-carbohydrate drinks worsening initial presentation?. Pediatr Diabetes.

[18] Kuppermann N, Ghetti S, Schunk JE (2018). Clinical trial of fluid infusion rates for pediatric diabetic ketoacidosis. N Engl J Med.

